# Distribution of Medals in an Auxiliary Hospital

**Published:** 1917-12-22

**Authors:** 


### Distribution of Medals in an Auxiliary Hospital.

An interesting ceremony was held recently in one of the military wards of the Leicester Royal Infirmary, when Surgeon-General Bedford of the Northern Command, who had been visiting the military hospitals in Leicester, attended to decorate three of the patients for heroic services.

The Mayor of Leicester having referred to the honour it was to have Surgeon-General Bedford among them, the General made the following presentations:

To Sergeant Bourget, 1st Canadian Mounted Infantry, the Military Medal for conspicuous bravery in carrying under heavy fire a wounded man across No Man's Land to his own dug-out, having first afforded first aid.

To Gunner H. Beck, a Leicester man, of the 46th North Midland Division Ammunition Column, the Military Medal for conspicuous gallantry in carrying a wounded comrade under very heavy shell-fire, having been wounded in the leg in performing this gallant act.

To Private N. R. Morris, 18th London Regiment, a similar medal for taking, when employed as Company Runner, an important message through heavy barrage fire; on another occasion he cut the German telephone wire, also under very heavy fire. The General also intimated that the gallant soldier had also been recommended for the Distinguished Conduct Medal.

To each soldier the General offered his hearty congratulations, and it was a real joy to be present to see the hearty and enthusiastic reception which the three heroes received from their comrades, who were all gathered in one ward for the ceremony.

